# A new notosuchian crocodyliform from the Early Palaeocene of Patagonia and the survival of a large-bodied terrestrial lineage across the K–Pg mass extinction

**DOI:** 10.1098/rspb.2024.1980

**Published:** 2025-03-26

**Authors:** Gonzalo Gabriel Bravo, Diego Pol, Juan Martín Leardi, Javier Marcelo Krause, Cecily S. C. Nicholl, Guillermo Rougier, Philip D. Mannion

**Affiliations:** ^1^ Instituto Superior de Correlacion Geologica, Yerba Buena, Argentina; ^2^ Museo Argentino de Ciencias Naturales Bernardino Rivadavia, Buenos Aires, Argentina; ^3^ Instituto De Estudios Andinos Don Pablo Groeber, Buenos Aires, Argentina; ^4^ Universidad de Buenos Aires Departamento de Biodiversidad y Biología Experimental, Buenos Aires, Argentina; ^5^ Museo Paleontológico Egidio Feruglio, Trelew, Argentina; ^6^ Universidad Nacional de Río Negro, Rio Negro, Argentina; ^7^ Department of Earth Sciences, University College London, London, UK; ^8^ Department of Anatomical Sciences and Neurobiology, University of Louisville, Louisville, KY, USA

**Keywords:** biogeography, body size, extinction selectivity, Salamanca Formation, Sebecoidea, Sebecidae

## Abstract

Sebecid notosuchians are the only terrestrial crocodyliforms to survive the Cretaceous–Palaeogene extinction, 66 Ma, which eliminated large-bodied species (above approximately 5 kg) in terrestrial ecosystems. Early sebecid evolution is unclear due to the scarcity of remains from both sides of the boundary. We present the stratigraphically earliest post-extinction notosuchian record, from the lower Palaeocene Salamanca Formation of Patagonia. *Tewkensuchus salamanquensis* n. gen. n. sp. has unique features, including a skull roof with elevated lateral margins, and an accessory peg and socket articulation between the postorbital and posterior palpebral. Our phylogenetic analysis allies *Tewkensuchus* with a clade of predatorial crocodyliforms from the Eocene of Europe (and possibly of Africa, as *Eremosuchus* may also belong to this clade). This clade forms the sister taxon of South American sebecids. We name Sebecoidea for this more inclusive clade of Eurogondwanan notosuchians and suggest that its spatial distribution reflects earlier diversification and dispersal events, which are only partially known. We estimate a body mass of around 300 kg for *Tewkensuchus*, one of the largest known notosuchians. Phylogenetic optimization of notosuchian body size change reconstructs a Cretaceous–Palaeogene boundary-crossing sebecoidean lineage with an estimated mass between 332 and 443 kg. This provides the first support for the survival of a large-bodied terrestrial vertebrate lineage across the K–Pg boundary.

## 1. Introduction

The Cretaceous-Palaeogene (K–Pg) mass extinction, 66 million years ago, was characterized by catastrophic biodiversity losses in both terrestrial and marine environments, whereas freshwater systems seemingly showed greater resilience [1–3]. This pattern is exemplified by the extinction of vertebrates with body masses exceeding approximately 5 kg in terrestrial ecosystems [4–6], including all non-avian dinosaurs and pterosaurs, as well as the loss of nearly all large-bodied marine reptiles [7], contrasting with the survival of some semi-aquatic and aquatic lineages in freshwater ecosystems that included large-bodied taxa, such as species of crocodyliforms and turtles [8,9].

Although the survival of small-bodied (<5 kg) birds, mammals and lepidosauromorphs within terrestrial ecosystems is frequently discussed, the notosuchian lineage of terrestrial crocodyliforms also survived the mass extinction event. Notosuchia was the most morphologically diverse clade of crocodyliforms at the time, with much of this diversity recorded in the Cretaceous of Gondwana [10–13]. Sebecidae and its close relatives form the only lineage of notosuchians to survive into the Cenozoic, with representatives mainly known from South America. Sebecids have been interpreted as primarily terrestrial predators that thrived in the early Cenozoic, prior to their extinction in the Middle Miocene [14–17]. Additionally, several predatorial taxa known from fragmentary remains in the Eocene of Europe and Africa have long been recognized as closely related to sebecids [17–24]. Until recently, unequivocal remains of sebecids and their close relatives were unknown from the Cretaceous, limited to fragmentary specimens with ziphodont dentition from the latest Cretaceous of Europe, which could not be confidently assigned to the group [21,24]. However, more recently, a partial skeleton from the Maastrichtian of Spain, representing the genus *Ogresuchus*, has been hypothesized as a member of this lineage [15].

The evolution and diversification of this group are particularly interesting, not only because it is the only lineage of terrestrial crocodyliforms [11,25] that survived the K–Pg mass extinction, but also because of the large body sizes that members of this group attained. For example, *Barinasuchus arveloi*, from the Middle Miocene of South America, is estimated to have exceeded six metres in body length and weighed more than a tonne [16,26]. However, the origins and phylogenetic affinities of Sebecidae and closely related taxa are still uncertain because the Cretaceous and Palaeocene remains of this lineage are scarce and typically fragmentary [15,27,28], and there is a lack of consensus among systematic studies. Phylogenetic analyses place sebecids either as the sister group of Baurusuchia (i.e. the Sebecosuchia hypothesis; [11,27,29,30]) or forming a clade with Peirosauridae, Itasuchidae and Mahajangasuchidae (i.e. the Sebecia hypothesis; [31–35]). Furthermore, in contributions that recover Sebecosuchia as monophyletic, the European Eocene species are depicted as sister taxa to Sebecidae [11,15,27,29], whereas some or all of these species are nested within Sebecidae in most studies that recover the sebecian topology [33–36]. These problems also mean that body size evolution in the group is poorly understood, including when large sizes are attained, further limiting our comprehension of the group’s survival across the K–Pg mass extinction event.

Here we describe a new crocodyliform that represents the earliest known notosuchian recorded after the K–Pg extinction event. Its age is well-constrained as it was found in the lower Palaeocene beds (~63.2−63.8 Ma [37]) of the Salamanca Formation, in the Punta Peligro area of Chubut Province, Argentinean Patagonia, which has previously yielded only caimanine crocodylian representatives of Crocodyliformes [38–40]. The new taxon differs markedly from caimanines due to its amphicoelous vertebrae, cranial ornamentation pattern and presence of a posterior palpebral, among other features. We analyse the phylogenetic affinities of the new taxon and show its relationship with taxa just outside of the sebecid radiation and discuss its implications for the biogeographic history of the group. Body mass estimates of the new taxon and related sebecids demonstrate, for the first time, the survival of large-bodied terrestrial vertebrates across the K–Pg mass extinction event.

## 2. Material and methods

### (a) Phylogenetic analysis

In order to test the affinities of the new taxon, we expanded the phylogenetic morphological matrix published by [27], which includes a large sample of ‘basal’ crocodyliforms and non-eusuchian mesoeucrocodylians; it represents the most recent iteration of the matrix originally presented by [29]. We expanded this matrix in terms of both taxon and character sampling, including the incorporation of five previously published taxa (*Eremosuchus*, *Doratodon ibericus*, *Doratodon carcharidens*, *Ogresuchus* and *Dentaneosuchus*) that have been depicted as closely related to Sebecidae in recent contributions [15,17,24]. *Sahitisuchus fluminensis* was excluded from our dataset because new specimens with important anatomical information are under description (A. Pinheiro 2023, personal communication). The resultant dataset was analysed using equally weighted parsimony in TNT v1.6 [41] with the New Technology search. The search was executed using sectorial searches, drift and tree fusing and stopping the search when the optimal tree was found 30 times. Then, a final round of TBR was performed. The most parsimonious trees (MPTs) were summarized using strict consensus, and unstable taxa were identified through the IterPCR script [42] to produce a well-resolved reduced strict consensus tree. We tested the effect of taxon instability using the script pcrjak [43] (see electronic supplementary material, data S1 for further details). The clade names and definitions used for Notosuchia follow those proposed by Leardi *et al*. [44] and the taxonomic nomenclature used by [27] for the *Sebecus* species.

### (b) Body size proxies

Body size and mass for notosuchian species were estimated using cranial measurements, following the approach of recent studies [45–47] that employ scaling equations based on cranial length of extant crocodylians [45,48–50] to estimate body length and mass in extinct crocodyliforms. We utilized measurements of the total dorsal cranial length (DCL) and dorsal orbito-cranial length (ODCL) in notosuchians as proxies because: (i) these have been shown to be good correlates of total body length and mass for extant crocodylians [48]; (ii) these can be used in a much larger number of extinct taxa than proxies based on postcranial measurements [46]; and (iii) preserved skeletons of sebecoid outgroups, such as *Araripesuchus gomesii*, *Alligatorellus beaumonti* and *Protosuchus richardsoni*, show similar DCL-to-body length proportions to extant crocodylians. Based on the above and given the current lack of complete skeletons among sebecoids, we consider it reasonable to assume for the moment similar skull-to-body size proportions in Sebecoidea and other notosuchians. We note however, that these estimates are used as rough approximations to enable general comparisons and identify major changes in the evolution of body size within Notosuchia.

We expanded the sample from the dataset used in [46] and [47], incorporating new Palaeogene data, particularly focusing on sebecids, to allow a more comprehensive analysis of body size evolution in Notosuchia from the Cretaceous to the early Neogene. To estimate dorsal cranial length (DCL) for incomplete sebecoid skulls, we used a regression model developed from data on five sebecids with complete skulls. This model, which shows that frontal bone length is a good predictor for DCL, was applied to estimate DCL in fragmentary sebecoid taxa, including *Tewkesuchus* (see electronic supplementary material, data S1 and S2).

### (c) Optimization of body mass across the K–Pg boundary

In order to evaluate notosuchian body size evolution, DCL values and body mass estimates were input in a data matrix as continuous characters. These estimates derived from [46,47], and the new estimates presented in this study (see electronic supplementary material, data S2), were then optimized onto the MPTs retrieved from the morphological phylogenetic analysis using the continuous optimization method implemented in TNT v1.6 [41]. This step allows for a refined examination of body mass evolution across the K–Pg boundary, taking into account newly added data. The optimization showing the increase in body mass across the K–Pg boundary is presented below, while the ancestral body mass reconstruction for each node is presented in the electronic supplementary material, data S1, figures S14–S15.

#### (i) Institutional abbreviations

AMNH FARB, American Museum of Natural History, Fossil Amphibians, Reptiles, and Birds section, New York, USA; MHNC, Museo de Historia Natural de Cochabamba, Cochabamba, Bolivia; MPEF, Museo Paleontológico Egidio Feruglio, Trelew, Argentina; PVL, Colección de Paleontología de Vertebrados Lillo, Tucumán, Argentina; STUS, Colección de Vertebrados Fósiles de la Cuenca del Duero (formerly ‘Sala de las Tortugas’), Universidad de Salamanca, Salamanca, Spain.

## 3. Results

### (a) Systematic palaeontology

CROCODYLIFORMES [51] (*sensu* [52])

MESOEUCROCODYLIA [53]

NOTOSUCHIA [14] (*sensu* [54])

SEBECOIDEA [55] [this work] nomen cladi conversum

Genus *TEWKENSUCHUS* nov.

*LSID*. urn:lsid:zoobank.org:pub:5AFE25CD-DD6D−4248−919D-D1C112A3B519

*Type species. Tewkensuchus salamanquensis* gen. et sp. nov.

*Derivation of name*. 'Tewken' derives from *t'ewk'en* or *t'ewq'en*, which means forehead in the language of the Tehuelche native people from southern Chubut. The generic name refers to the frontal bone of the type specimen, which shows numerous unique features among notosuchians. 'Suchus' derives from the Greek *Σοῦχος* (Soukhos, Souchos), an Egyptian crocodile-headed god.

*Diagnosis*. Notosuchian crocodyliform that differs from other members of the clade by the following unique combination of characters (autapomorphies marked with an asterisk): (1) pattern of ornamentation on the dorsal surface of the frontal changing from well-marked subparallel grooves on the anterior region to shallower grooves that vary in their length and orientation on the posterior region; (2) lateral margin of the frontal forming an almost right angle with the posterior region of orbit, expanding abruptly towards its contact with the postorbital; (3) presence of a distinctive depression at the contact between the frontal and postorbital at the posteromedial corner of the orbit; (4) skull roof with dorsal surface of the postorbital elevated relative to that of the frontal*; (5) vertically oriented sutural contact on the postorbital for the posterior palpebral*; (6) accessory peg and socket articulation between postorbital and posterior palpebral located at the ventral end of their sutural contact*; (7) hypertrophied conical teeth with smooth, non-serrated carinae; (8) carinae restricted to apical half of tooth crowns; (9) ventral surface of the cervical vertebrae smooth, lacking a keel.

*Tewkensuchus salamanquensis* gen. et sp. nov.

*LSID*. urn:lsid:zoobank.org:act:

*Derivation of name*. s*alamanquensis* derives from the name of the formation from which the type specimen comes (i.e. the Salamanca Formation).

*Type specimen*. MPEF-PV 3700, fragmentary skull, comprising tooth-bearing fragments, prefrontal, frontal, postorbitals, right anterior, and two posterior palpebral bones, isolated tooth, cervical and caudal vertebrae, and phalanges (figures 1 and 2; electronic supplementary material, figures S3–S8).

**Figure 1.**
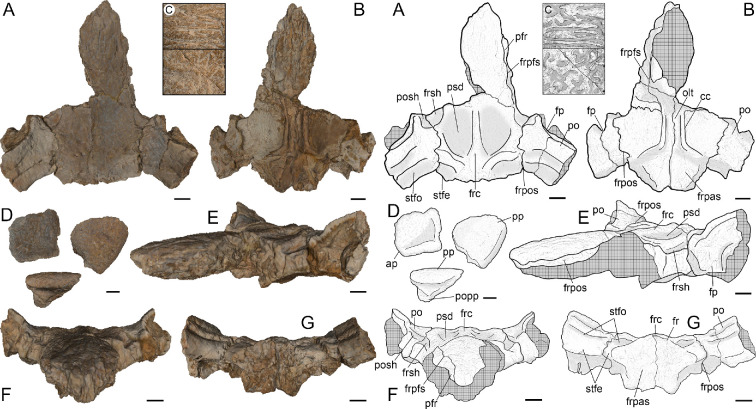
Image and drawing of the 3D model of *Tewkensuchus salamanquensis* gen. et sp. nov. (A) Dorsal view of skull elements. (B) Ventral view of the skull elements. (C) Detail of the ornamentation on the anterior (up), and posterior region (down) of the frontal. (D) Anterior palpebral in dorsal view (up to the left) and posterior palpebral in dorsal (up to the right) and posterior views (down). (E) Anterolateral view. (F) Anterior view. (G) Posterior view. Anatomical abbreviations: ap, anterior palpebral; cc, crista cranii; fp, facet for posterior palpebral articulation; fr, frontal; frc, frontal crest; frpas, frontal-parietal articular surface; frpos, frontal-postorbital suture; frpfs, frontal-prefrontal suture; frsh, frontal shelf; stfe, supratemporal fenestra; stfo, supratemporal fossa; olt, olfactory tract; pfr, prefrontal; po, postorbital; popp, postorbital contact area with the posterior palpebral; posh, postorbital shelf; pp, posterior palpebral; psd, parasagittal depression. Scale bar represents 10 mm.

**Figure 2.**
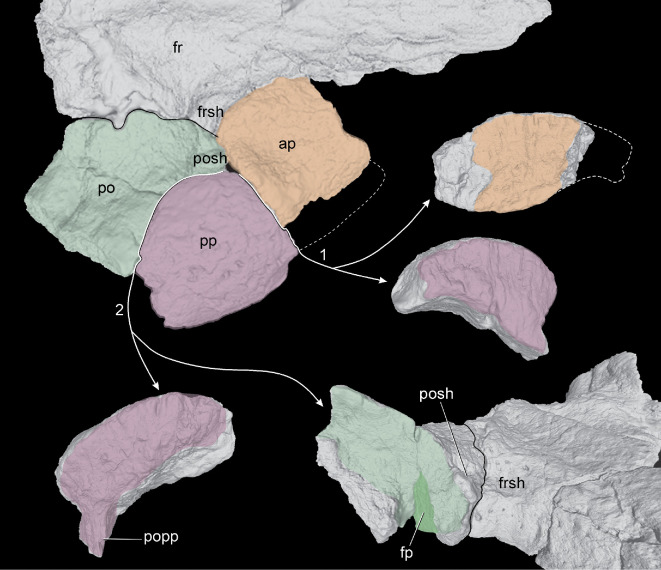
Location of the palpebrals on the cranial roof of *Tewkensuchus salamanquensis* gen. et sp. nov. in dorsal (top left) and right anterolateral (bottom right) views; green (postorbital), purple (posterior palpebral), and orange (anterior palpebral) colors indicate three elements of the cranial roof; arrow 1, anterior and posterior palpebral suture; arrow 2, suture between postorbital and posterior palpebral; the bifurcation of the arrows show the sutural surface of both elements; black line, postorbital-frontal interdigitated suture; interrupted white line, reconstructed area of the partially preserved anterior palpebral. Anatomical abbreviations: ap, anterior palpebral; fr, frontal; fp, facet (socket) for posterior palpebral peg; po, postorbital; popp, posterior palpebral peg on contact surface with the postorbital; pp, posterior palpebral. Images not to scale.

*Diagnosis*. Same as for the monotypic genus.

*Occurrence*. Punta Peligro area, 45 km north of Comodoro Rivadavia City, Chubut Province, Argentina (45° 30′ 49.1″ S, 67° 15′ 36.91″ W). Salamanca Formation [56], Danian, lower Palaeocene (see Geological setting; electronic supplementary material, data S1).

### (b) Description and comparisons

*Skull roof*. The preserved elements include the articulated frontal, postorbitals, the right anterior palpebral, both posterior palpebrals, and a small non-informative fragment of a prefrontal. The frontals are fully fused (figure 1), as in all adult mesoeucrocodylian crocodyliforms [52]. The dorsal surface has two mediolaterally broad parasagittal depressions (psd; figure 1), separated by a well-developed sagittal crest. This morphology is present in *Iberosuchus* (STUS-13623 [57]), *Dentaneosuchus* [17], and some baurusuchids (e.g. *Pissarrachampsa*, *Aphaurosuchus*, *Baurusuchus*, *Stratiotosuchus* and *Aplestosuchus*), among other notosuchians. The frontal crest of *Tewkensuchus* is high and broad (figure 1), as in *Iberosuchus* (STUS−13623) and *Dentaneosuchus* [17]. However, this morphology contrasts with that of sebecids, in which the crest is consistently narrower (i.e. ridge-like), being lower in *Sebecus icaeorhinus* (AMNH FARB 3160), and higher in S*ebecus querejazus* (MHNC-P 3701) and the Lumbrera form (PVL 6385).

Anteromedial to the contact with the postorbital, at the posteromedial corner of the orbit, the frontal bears a small shelf ventrally recessed relative to its dorsal surface. This distinctly depressed surface is approximately semicircular (frsh; figure 1A,F) and continues onto the anteromedial corner of the postorbital (posh; see below). The surface of this depressed shelf is smooth and resembles the shelf on the postorbital of other notosuchians for the posterior palpebral (e.g. peirosaurids, sphagesaurians, uruguaysuchids) [32,35], but in *Tewkensuchus,* this shelf is not overlapped by a palpebral (figure 2). Among crocodyliforms, the presence of this distinctive depression on the frontal is a feature otherwise known only in *Iberosuchus* sp. (STUS-13623 [57]). Anterior to this depression, the orbital margin of the frontal of *Tewkensuchus* lacks any signs of an articular surface for a palpebral.

The postorbital bears unique features for a crocodyliform. First, with the dorsal surface of the frontal held horizontally, the dorsal surface of both postorbitals are inclined such that they face dorsomedially (figure 1E–G). Therefore, the lateralmost region of the skull roof is elevated relative to the rest of the skull table. This contrasts with the flat and horizontal skull table of most crocodyliforms [29], and is therefore regarded as an autapomorphy of *Tewkensuchus*.

The anterior surface of the postorbital also bears unique characteristics. There is a large vertical facet that faces anterolaterally and occupies most of the anterior surface that is sutured with the posterior palpebral (figure 2). This facet of the postorbital is dorsoventrally high, slightly concave, and articulates with a slightly convex facet of the posterior palpebral (fp; figure 2). At the ventromedial end of this contact, the sutural surface bears an accessory peg of the palpebral that fits into a narrow socket of the postorbital (popp; figure 2). This type of contact is also unique among crocodyliforms.

**Figure 3.**
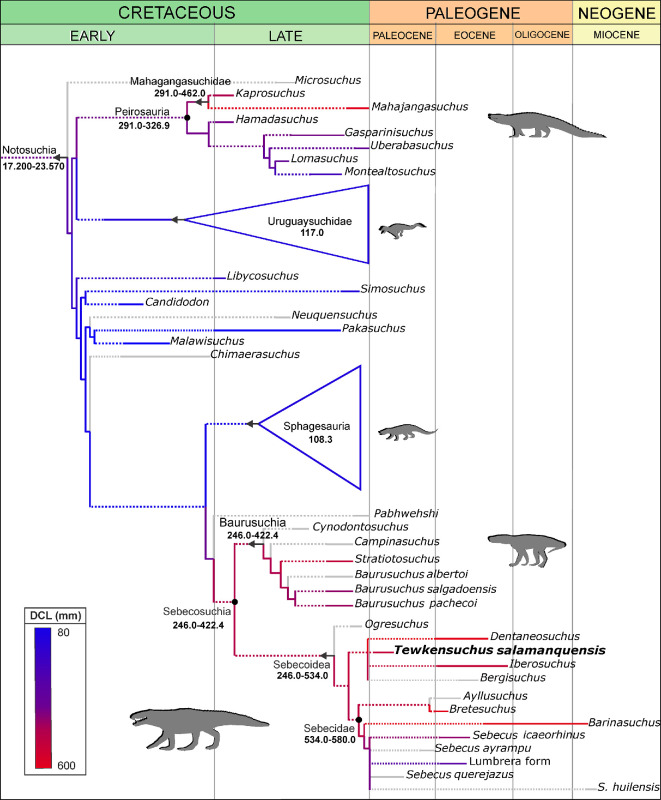
Parsimony optimization of cranial length (DCL) across the evolution of Notosuchia. The DCL values were optimized as a continuous character in TNT. The colour scale represents from blue (lowest DCL values) to red (largest value DCL value) the progressive increases in DCL (proportional to body mass) across the topology (see electronic supplementary material, data S2, for further details). The time-calibrated topology is based on the reduced strict consensus of our phylogenetic analysis. Dashed lines in terminal branches indicate inferred ghost lineages and solid lines along terminal branches indicate stratigraphic uncertainty. The age ranges for each taxon were extracted from the Paleobiology Database (https://paleobiodb.org). The different symbols on the nodes indicate the definitions followed for those clades (arrows, maximum clade definitions; dots, minimum clade definitions), while the ranges beneath the clade names show the optimization for each of them.

The medialmost region of the anterior surface of the postorbital has a smooth, distinct depression at the orbital margin (posh; figures 1A and 3). This depression is the lateral continuation of the aforementioned depression on the frontal, a morphology shared with *Iberosuchus*.

Three palpebrals are preserved (figure 1D) and two represent a posterior palpebral. The anterior two-thirds of the right anterior palpebral are preserved. This fragment contacts the prefrontal, frontal, a small area of the postorbital, as well as the posterior palpebral (figure 2). The dorsal surface of the anterior palpebral is subquadrangular (although it is broken, due to fracture), whereas the posterior palpebral is subtriangular (figure 1D). Ventrally, the posterior palpebral has a wedge-shaped peg. For a more detailed description and figures of the skull roof, see the electronic supplementary material, data S1.

*Dentition*. The conical crown is characterized by convex labial and lingual surfaces, lacking the strong labiolingual compression of some sebecids. Its mesial and distal edges form continuous and sharp but non-serrated carinae, and the enamel surface is smooth (see electronic supplementary material, data S1 for the figure of the tooth, as well as details and comparison of the dentition).

*Postcranial skeleton*. Partial remains of six cervical and eight caudal vertebrae are preserved. These vertebrae are amphicoelous and their remains are heavily damaged, but, nonetheless, it is possible to identify their corresponding region in the vertebral column based on the morphology of the vertebral centra. Fragments of 14 phalanges are preserved, six of which are ungual phalanges. It is not possible to confidently determine whether they correspond to the manual or pedal autopodium, or to identify their digit number and position. For a more detailed description and figures of the postcranial elements, see the electronic supplementary material, data S1.

### (c) Phylogenetic relationships

Anatomical features of the skull and postcranium indicate that *Tewkensuchus salamanquensis* is a new non-eusuchian mesoeucrocodylian, with affinities to sebecid notosuchians and closely related taxa (figure 3). Our phylogenetic analysis results in more than 10 000 MPTs of 1768 steps (CI = 0.302, RI = 0.742) that produce a reduced strict consensus (obtained by IterPCR script) that is largely congruent with topologies emanating from previous iterations of this data matrix [11,13,15,27,30,58]: Sebecidae and its close relatives cluster with Baurusuchia, thus supporting the monophyly of Sebecosuchia (figure 3). This clade is supported by two unambiguous synapomorphies: dorsal surface of mandibular symphysis strongly concave and narrow, trough-shaped (char. 189.1); and presence of circular paramedian depressions located anteriorly on the palatal surface of the premaxilla (char. 227.1). The support values for Sebecosuchia are relatively low (pcrjack values below 70%; electronic supplementary material, figure S11). However, forcing the monophyly of Sebecia in this dataset results in trees that are 22 steps longer than the MPTs of the unconstrained search.

All MPTs depict *Tewkensuchus salamanquensis* in a clade with taxa from the Eocene of Europe: *Bergisuchus*, *Dentaneosuchus* and *Iberosuchus* (figure 3). Its inclusion in this clade is supported among other features by derived similarities in the frontal of *Tewkensuchus* and *Iberosuchus*: (i) a distinct depressed surface on the frontal at the posteromedial corner of the orbit (char. 442.1); (ii) lateral margin of the frontal forming an almost right angle with the posterior region of orbit, expanding abruptly towards its contact with the postorbital (char. 444.1). The three unambiguous synapomorphies that diagnose this clade in all the MPT are however either unknown (chars. 161.1 and 315.1) or modified (char. 353.2) in *Tewkensuchus* (electronic supplementary material, data S1). In some of the MPTs the fragmentary taxon *Eremosuchus elkoholicus* [19] from the Eocene of Africa is allied with this clade (electronic supplementary material, figure S12). This monophyletic group of Eurogondwanan species is the sister clade to Sebecidae in all MPTs. The latter includes *Barinasuchus*, along with two main monophyletic groups within this family: *Ayllusuchus + Bretesuchus*, and the *Sebecus* clade (*sensu* [27]), which includes all species of *Sebecus* and the ‘Lumbrera Form’. Although *S. querejazus* is detected as unstable among the MPTs (electronic supplementary material, figure S12; figure 3), the alternative positions of this species are restricted within the *Sebecus* clade.

Our phylogenetic analysis retrieves a clade formed by Sebecidae and several species from the Late Cretaceous–Palaeogene of Eurogondwana [15,17,24], which is here named Sebecoidea (figure 3; see definition in table 1). Sebecoidea is diagnosed by two unambiguous synapomorphies: (i) external surface of maxilla forms a single, laterally facing plane (char.139.0); and (ii) distal half of tibial shaft posteriorly bowed (char. 335.1). Sebecoidea is also characterized by 28 ambiguous synapomorphies (see electronic supplementary material, data S1). Support values for Sebecoidea, ignoring unstable taxa detected by pcrjak are high and reach up to 84% (electronic supplementary material, figure S11), although one of the taxa detected as unstable by this method is *Tewkensuchus salamanquensis*.

**Table 1.** New phylogenetic definition proposed for the clade discussed in this study.

lade name and registration	phylogenetic definition, reference phylogeny and composition
Sebecoidea Simpson, 1937 [this work], converted clade name Registration number: 1088	phylogenetic definition: The most inclusive group that contains *Sebecus icaeorhinus* Simpson, 1937, *Iberosuchus macrodon* Antunes, 1975 and *Bergisuchus dietrichbergi* Kuhn, 1968, but not *Baurusuchus pachecoi* Price, 1945, *Mahajangasuchus insignis* Buckley and Brochu, 1999, *Montealtosuchus arrudacamposi* Carvalho, de Vasconcellos, and Tavares, 2007, *Peirosaurus torminni* Price, 1955, *Notosuchus terrestris* Woodward, 1896, and *Crocodylus niloticus* Laurenti, 1768. This is a maximum clade definition. Reference phylogeny: Phylogenetic hypothesis depicted in figure 3 of this work. In the reference phylogeny Sebecoidea is the sister group of Baurusuchia (supporting sebecosuchian monophyly). Other studies [31,33,35,36,54] depict sebecoids as the sister group of either Mahajangasuchidae or Peirosauridae (supporting sebecian monophyly). Composition: Sebecoidea includes Sebecidae and some species-level taxa. These taxa (*Iberosuchus* and *Bergisuchus*) have been recovered forming a clade, as the sister group of Sebecidae, in recent contributions [15,27].

### (d) Body mass estimation and optimization

Although DCL cannot be measured in the known remains of *Tewkensuchus salamanquensis*, the anteroposterior length of the frontal and DCL are strongly positively correlated with the length of the skull in all known sebecid taxa (electronic supplementary material, data S2). Thus, using the length of the frontal, we estimate the DCL of *Tewkensuchus salamanquensis* to be approximately 520 mm. The anterior region of the frontal is nearly complete on the right side and we measured the length on this side. If we assume a similar relationship of skull length and body mass as in extant crocodylians (see table 3 in [45]), the estimated skull length of *Tewkensuchus* would imply a body mass of ~300 kg, placing it with a higher estimate than the largest known baurusuchid, *Stratiotosuchus maxhechti*, at almost ~280 kg, and the larger estimates for peirosaurians (*Kaprosuchus saharicus*, *Stolokrosuchus lapparenti*), at approximately 230 kg. Paralleling the large morphological disparity in cranial anatomy of Sebecidae [27,59], we note that the range of body masses among sebecoideans is larger than that of other notosuchian clades (*Lorosuchus nodosus*, for example, would have reached approximately 23 kg, *Sebecus icaeorhinus* ~116 kg, and *Dentaneosuchus* ~773 kg; electronic supplementary material, data S2). The only other clade with notable body mass disparity among notosuchians is Peirosauria (e.g. *Montealtosuchus arrudacamposi* is ~12 kg, *Gasparinisuchus peirosauroides* is ~63 kg; electronic supplementary material, data S2).

### (e) Optimization of body mass on our phylogenetic topology

Mapping evolutionary change in our phylogenetic topology reconstructs a significant escalation in size from the base of Notosuchia, optimized between 6 and 25 kg (electronic supplementary material, figure S12 and data S2). Furthermore, this optimization reveals two independent positive progressive changes of body mass increase in Peirosauria and Sebecosuchia. The base of the Sebecosuchia node is optimized between 147 and 280 kg during the Late Cretaceous and there is an increase along the basal nodes of Sebecoidea so that the optimized body mass of the lineage that crosses the K–Pg boundary ranges between 332 and 443 kg (electronic supplementary material, figure S12 and data S2). Subsequently, there was a further increase in body mass during the Cenozoic, with *Dentaeosuchus* from the Eocene exceeding 700 kg and *Barinasuchus* from the Miocene of Venezuela reaching a mass of 500 kg (electronic supplementary material, figure S12 and data S2). Additionally, there were size reduction trends also reconstructed in the clade *Sebecus*, with the Lumbrera form and *S. ayrampu* having masses less than 30 kg, from the middle Eocene and middle–late Palaeocene of Argentina, respectively (see electronic supplementary material, data S2).

## 4. Discussion

### (a) Systematics of Sebecoidea

One of the non-sebecid taxa recovered in Sebecoidea in our analysis is *Iberosuchus*, known from several early–middle Eocene European localities [20,22,60]. The possible affinity of this taxon with sebecids has been recognized for decades, but it was only first depicted in a clade allying *Iberosuchus* with sebecids in a phylogenetic analysis by [61]. Subsequently, this result has been retrieved in most phylogenetic studies [11,27,62,63]. A second European taxon allied to sebecids in our results is *Bergisuchus* from the middle Eocene of Germany [23,64], a relationship that has also been supported in multiple phylogenetic analyses (e.g. [11,15,21,27,35,58,65–68]). Our results place the recently described *Ogresuchus*, from the early Maastrichtian of Spain, as the earliest branching member of Sebecoidea, being the sister taxon to the clade composed of Sebecidae and the *Bergisuchus+Dentaneosuchus+Iberosuchus+Tewkensuchus* clade. This represents a slightly more ‘basal’ position for *Ogresuchus* than that of the analysis of Sellés *et al*. [15], who retrieved it within Sebecidae. Nonetheless, heuristic tree searches constraining *Ogresuchus* within Sebecidae require only one extra step.

The position of *Dentaneosuchus* is similar to that of the original description by [17], although these authors retrieved it more basally than *Iberosuchus* and regarded all these taxa as part of a more inclusive conception of Sebecidae (equivalent to what is here referred to as Sebecoidea). Except for [17], all previous phylogenetic studies excluded the Palaeogene taxa of Europe from Sebecidae; therefore, we consider it more consistent with previous usage to keep a more restrictive conception of Sebecidae (as in [44]). The new clade name Sebecoidea applies to the more inclusive clade that includes *Ogresuchus*, the Eurogondwanan clade that groups *Bergisuchus*, *Dentaneosuchus*, *Iberosuchus* and *Tewkensuchus*, and the South American lineage Sebecidae (currently known only from the Palaeogene and early Neogene).

*Eremosuchus elkoholicus* [19] from the early Eocene of Algeria and two species of the genus *Doratodon* (*D. ibericus* and *D. carcharidens*), both from the latest Cretaceous of Europe [21,24,60,69,70], are also included in our phylogenetic analysis. These three taxa had been allied to sebecoid taxa by previous authors and could be particularly relevant for understanding the evolutionary history of Sebecoidea because *Doratodon* would be the stratigraphically oldest record of the group (Santonian of Hungary [24]), and *Eremosuchus elkoholicus* is the only African taxon that has been related to this group (along with fragmentary materials from the late Eocene of Egypt [71]). Whereas several studies have depicted *Doratodon* as close to Sebecidae [21,24,72], few studies have included *Eremosuchus* in a phylogenetic analysis, in which it has been recovered as close to sebecids or to baurusuchids [20,65,72]. Our analysis retrieves these three species as highly unstable taxa, probably as a result of being represented by very incomplete specimens (electronic supplementary material, figure S12). The alternative positions recovered for both *Eremosuchus elkoholicus* and *Doratodon ibericus* suggest a close relationship with Sebecosuchia, but it is unclear whether they belong to Baurusuchia or Sebecoidea, similar to what was reported in previous studies [21,24,65] (electronic supplementary material, figure S12). As noted above, *Eremosuchus elkoholicus* is recovered as an additional member of the *Bergisuchus+Dentaneosuchus+Iberosuchus+Tewkensuchus* clade in some MPTs, which would be consistent with the spatiotemporal distribution of the latter four species. On the other hand, *Doratodon carcharidens* is consistently recovered outside of Sebecosuchia, as in another recent study [54]. Our analyses suggest several alternative positions for *Doratodon carcharidens* within Mesoeucrocodylia, including its placement at the base of this clade, within Notosuchia, and even within Neosuchia. More detailed study of all three of these species will be required to determine their phylogenetic affinities.

### (b) Biogeographic implications for Sebecoidea

The presence of early diverging sebecoideans in the latest Cretaceous and early Palaeogene of Europe and South America, as we recovered here, potentially indicates an unusual palaeobiogeographic history (see also [15,24]). Given the absence of notosuchians from North America throughout the group’s evolutionary history [29], we can exclude this well-sampled continent as a potential dispersal route. Transoceanic dispersal also seems improbable given the distance involved and the primarily terrestrial habits inferred for sebecoideans. The most likely dispersal route involves Africa, with evidence for geologically ephemeral land bridges that connected it with Europe during some intervals of the Cretaceous and early Palaeogene (e.g. [24,73,74]). The complete separation between Africa and South America appears to have occurred in the mid-Cretaceous [75], although there is evidence of potential island stepping stones that later linked these two landmasses, allowing for trans-Atlantic dispersals in other terrestrial lineages [74,76]. One additional problem with this explanation is that we lack unequivocal evidence for sebecoideans in Africa, and probable occurrences of this clade (e.g. *Eremosuchus*) are restricted to the Eocene. However, the African terrestrial fossil record is scarce for much of the post-Cenomanian to Palaeocene and thus the absence of sebecoideans on this continent could represent sampling failure [13].

Representatives of Sebecosuchia date back to at least the Santonian, based on remains of South American baurusuchians (e.g. [77]), which means that sebecoideans must also have evolved by then as their sister clade (figure 3). Given the possibility that sebecosuchians were also present in Europe by the Santonian (i.e. *Doratodon*), this implies that the diversification and dispersal of the clade probably happened earlier and is currently unsampled, as has been proposed for other notosuchian lineages (e.g. [13]). Indeed, if the sebecosuchian affinities of the Middle Jurassic Malagasy crocodyliform *Razanandrongobe* are confirmed, as suggested in some some previous studies [15,63,77], then this suggests major spatiotemporal gaps in our sampling of notosuchian evolutionary and biogeographic history [47]. As such, it is possible that ancestral sebecoids dispersed between Africa and South America while these continents were still connected, and dispersals between Africa and Europe occurring during sea-level lowstands and emergent landbridges, as has been proposed for many dinosaur lineages and other non-marine tetrapods (e.g. abelisaurid theropods, rebbachisaurid sauropods, other notosuchians clades, madtsoiid snakes, podocnemidid turtles; [74]). This would explain the presence of *Tewkensuchus* in South America, *Ogresuchus* and multiple Eocene taxa in Europe, and *Eremosuchus* in Africa. An alternative scenario is that the first appearance of sebecoids in the fossil record might be closer to the timing of their origin (i.e. *Tewkensuchus* in the early Palaeocene of Argentina and *Sebecus querejazus* from the early–middle Palaeocene of Bolivia; [28]). Under this alternative scenario their origin may have occurred in South America, with subsequent dispersal events to Europe (via Africa) in the Cenozoic.

### (c) Body size extinction selectivity across the K–Pg boundary

Sebecoideans are intriguing partly because of their obscure origins, but also because they apparently developed diverse ecological habits [59]. This diversity provides an opportunity to study ecomorphological changes and the factors that may have influenced species survival in terrestrial ecosystems during the most recent mass extinction event. Extinction rates in freshwater aquatic environments were notably lower than in terrestrial ecosystems across the K–Pg boundary [2,3,78]. It has been estimated that no terrestrial animal larger than ~5−10 kg survived this extinction event [79–82]. Larger-bodied crocodyliforms that survived, comprising crocodylians, dyrosaurids and pholidosaurids, were partly or fully aquatic (e.g. [83]). Although some sebecoideans also survived the event, their representatives have been interpreted as primarily terrestrial large predators [11,25,58,84]. This sets them apart from most other notosuchian lineages that were smaller in body size and with diverse dietary habits [82,84–87], except for some peirosaurians and baurusuchians. Godoy *et al*. [46] proposed that body size increase over time in crocodyliforms might be due to evolutionary trends across multiple lineages, or selective extinction of small-bodied species. Studies exploring biotic and abiotic factors affecting notosuchians attribute their survival through the K–Pg event to a shift from small lineages with omnivorous diets to larger, hypercarnivorous ones during the Late Cretaceous [82,84]. Kellner *et al*. [58] suggested that sebecids were less specialized carnivores than baurusuchids, noting that the significant reduction in tooth number is indicative of a higher degree of specialization. Furthermore, Buffetaut [88] raised the possibility that only the less specialized crocodyliforms survived the K–Pg event.

Mapping skull length as a continuous character on our notosuchian phylogeny reveals a gradual increase in body size towards the end of the Cretaceous in two independent clades, Peirosauria and Sebecosuchia (figure 3). Several members of the latter clade, interpreted as hypercarnivorous terrestrial species [84], were already large predators by the latest Cretaceous, reaching close to 300 kg in some taxa (e.g. *Stratiotosuchus*) (see electronic supplementary material, figure S13 and data S2). Our optimization indicates that the K–Pg boundary-crossing sebecoid lineage had attained larger body sizes, estimated between 332 and 443 kg (electronic supplementary material, figure S14). Even the inclusion of a small body size estimate for *Ogresuchus* in our optimization does not affect the large body size inferred for the K–Pg crossing lineage. Therefore, we argue that the survival of a large body size lineage of terrestrial carnivores is the most likely scenario and could be a further indication that the global effects of the K–Pg bolide impact were less severe and recovered faster in the Southern Hemisphere relative to its northern counterpart [89–94].

The survival of a lineage of large-bodied terrestrial carnivores contradicts patterns of extinctions of all large vertebrates in terrestrial ecosystems [4–6]. However, two alternative scenarios could potentially reconcile sebecoid evolutionary history with hypotheses suggesting body size selectivity in survival across the K–Pg event. The first alternative scenario is the surviving sebecoid lineage could have originated from more aquatic forms and therefore be better equipped to forage in freshwater ecosystems, which experienced comparatively lower extinction rates [1]. This scenario may be plausible if sebecoids are indeed more closely related to peirosaurians [31,33,54]—some of which were interpreted as more aquatic than other notosuchians based on their anatomy [95–97]. The second possible scenario is that the sebecoid lineage that crossed the K–Pg boundary was indeed terrestrial, but composed of small bodied forms. This possibility arises from the remarkably scarce Cretaceous fossil record of sebecoideans, which is currently limited to *Ogresuchus*, a small bodied species (~1 m in length and ~9 kg in mass) from the latest Cretaceous of Europe, recovered here as the earliest diverging sebecoid. If future discoveries reveal additional Cretaceous sebecoids with similarly small body sizes, it is possible that the sebecoid lineage that survived the K–Pg event was primarily composed of small-bodied taxa. Under this scenario, the presence of the large-bodied *Tewkensuchus* in the early Palaeocene would imply a rapid increase in body size after the K–Pg event. Nevertheless, as noted above and based on the current fossil record, our phylogenetic analysis reconstructs that a large body size for the sebecoid lineage that crossed the K–Pg boundary (figure 3) is the most parsimonious scenario.

## 5. Conclusions

*Tewkensuchus salamanquensis* n. gen. n. sp. increases the diversity of Palaeogene South American crocodyliforms at high latitudes, in particular from the Palaeocene of Patagonia, and represents the earliest known post-K–Pg notosuchian with a chronostratigraphically well-constrained age. The inclusion of *Tewkensuchus* and other sebecosuchian taxa (e.g. *Doratodon ibericus*, *Ogresuchus furatus*, *Dentaneosuchus crassiproratus*, *Eremosuchus elkoholicus*) in our phylogenetic analysis provides a framework for establishing a phylogenetic definition for the new clade Sebecoidea, which is consistent with previous contributions [11,15,27,35,58,66,68], and restricts currently known members of Sebecidae to taxa recorded in the Palaeogene and early Neogene of South America. The preserved remains of *Tewkensuchus salamanquensis* contribute to expanding the known morphological diversity (e.g. dentition and cranial roof structure) among early non-sebecid sebecoideans, further distinguishing them from other notosuchians. The stratigraphic age of *Tewkensuchus* and its close relationship with European Eocene taxa indicate a complex history of early diversification and dispersal events of sebecoids that is yet to be fully understood. The analysis of the evolution of body size in Notosuchia, particularly in Sebecoidea, reveals patterns that do not conform to current assumptions about the fate of large land vertebrates across the K–Pg extinction event, suggesting the survival of a large-bodied lineage that evolved before this catastrophic event. This challenges previous ideas and enriches our understanding of evolutionary dynamics during this time.

## Data Availability

Data for this study (e.g. additional descriptions, character argumentations, phylogenetic datasets, phylogenetic results, additional figures) are available in the Dryad Digital Repository: https://doi.org/10.5061/dryad.ncjsxkt4z. The three-dimensional model of the holotype of Tewekensuchus salamanquensis is available in the MorphoSource repository: https://www.morphosource.org/concern/media/000716298?locale=enhttps://www.morphosource.org/concern/media/000716298?locale=en. Supplementary material is available online [98].
